# Identifying effective connectivity parameters in simulated fMRI: a direct comparison of switching linear dynamic system, stochastic dynamic causal, and multivariate autoregressive models

**DOI:** 10.3389/fnins.2013.00070

**Published:** 2013-05-14

**Authors:** Jason F. Smith, Kewei Chen, Ajay S. Pillai, Barry Horwitz

**Affiliations:** ^1^Brain Imaging and Modeling Section, National Institute on Deafness and Other Communication Disorders, National Institutes of HealthBethesda, MD, USA; ^2^Positron Emission Tomography Center and Banner Alzheimer's Disease Institute, Banner Good Samaritan Medical CenterPhoenix, AZ, USA; ^3^Department of Mathematics and Statistics, Arizona State UniversityTempe, AZ, USA; ^4^Arizona Alzheimer's Disease ConsortiumPhoenix, AZ, USA; ^5^Human Motor Control Section, National Institute on Neurological Disorders and Stroke, National Institutes of HealthBethesda, MD, USA

**Keywords:** effective connectivity, fMRI BOLD, modeling and simulation, parameter estimation

## Abstract

The number and variety of connectivity estimation methods is likely to continue to grow over the coming decade. Comparisons between methods are necessary to prune this growth to only the most accurate and robust methods. However, the nature of connectivity is elusive with different methods potentially attempting to identify different aspects of connectivity. Commonalities of connectivity definitions across methods upon which base direct comparisons can be difficult to derive. Here, we explicitly define “effective connectivity” using a common set of observation and state equations that are appropriate for three connectivity methods: dynamic causal modeling (DCM), multivariate autoregressive modeling (MAR), and switching linear dynamic systems for fMRI (sLDSf). In addition while deriving this set, we show how many other popular functional and effective connectivity methods are actually simplifications of these equations. We discuss implications of these connections for the practice of using one method to simulate data for another method. After mathematically connecting the three effective connectivity methods, simulated fMRI data with varying numbers of regions and task conditions is generated from the common equation. This simulated data explicitly contains the type of the connectivity that the three models were intended to identify. Each method is applied to the simulated data sets and the accuracy of parameter identification is analyzed. All methods perform above chance levels at identifying correct connectivity parameters. The sLDSf method was superior in parameter estimation accuracy to both DCM and MAR for all types of comparisons.

## Introduction

There is a growing consensus that cognition can be fruitfully analyzed by studying the dynamic integration of information computed by multiple, spatially discrete, functionally specialized brain regions (McIntosh, 1999, 2001). In studies of cognition utilizing functional magnetic resonance imaging (fMRI), as well as other neuro-imaging methods, application of functional and effective connectivity measures has become dramatically more common. Connectivity methods are used to identify approximations to the integrative dynamics of multiple brain regions at a coarse-grained level of analysis. For fMRI, the spatial scale is measured in millimeters and the temporal scale measured in seconds. Though, these scales are far from that of neurons, dendrites, and spikes, theory, and evidence strongly suggests that the dynamic patterns of interactions at any level of analysis can be analyzed roughly independently from the details of the smaller units that interact to form the patterns themselves (Kelso, 1995; Haken, 1996). Thus, adequate understanding of the dynamic interactions between brain regions measured at the scale of fMRI may not require complex units such as multi-compartment spiking neurons as a computational substrate. The interregional interactions at this coarser level are potentially interesting in their own right and represent a rich dataset to mine for unique biomarkers of disease and differential abilities (Büchel et al., 1999; Mechelli et al., 2002; Bokde et al., 2009; Whalley et al., 2009; Rowe, 2010; Horwitz and Rowe, 2011).

Increased interest in connectivity analysis of neuro-imaging has resulted in an explosion of the number of different methods for estimating the existence and strength of interregional interactions (Moeller and Strother, 1991; Horwitz et al., 1992; Alexander and Moeller, 1994; Friston, 1994; McIntosh and Gonzalez-Lima, 1994; Bullmore et al., 1996; McIntosh et al., 1996; McKeown et al., 1998; Büchel et al., 1999; Friston et al., 2003; Harrison et al., 2003; Sun et al., 2004; Valdés-Sosa, 2004; Roebroeck et al., 2005, 2011; Seth, 2005; Valdés-Sosa et al., 2005; Marrelec et al., 2006; Patel et al., 2006; Shimizu et al., 2006; Smith et al., 2006, 2010; Rajapakse and Zhou, 2007; Havlicek et al., 2010; Chen et al., 2011). Each connectivity method approximates interregional brain dynamics in a specific manner as determined by the statistical model behind the method. Though the term “connectivity” is consistently used to describe each of these methods, the underlying statistical models are often not consistent. Because of these differences, it is not clear which methods are directly comparable or even attempting to measure the same underlying type of approximate dynamics (Horwitz, 2003). How then should researchers evaluate different connectivity methods and select the method appropriate to their study? If one method identifies a pattern of connectivity in a dataset that another method fails to find does this indicate the first method is superior (cf. David et al., 2008)? If two methods identify a similar pattern of connectivity in the same dataset, does this indicate the connectivity is more likely to exist (cf. Penny et al., 2004)? Answers to these questions will require a deeper understanding of the relations between the mathematical foundations of different connectivity measures and direct comparative evaluations designed with these relations in mind.

The most direct comparison for connectivity methods is relative accuracy. That is, can the method identify interactions between functional units at the correct magnitude? This direct test requires knowledge of the ground truth; the nature, location, and magnitude of the actual interregional interactions. Unfortunately, this ground truth is simply unknown for *in vivo* data for several reasons. To avoid this difficulty, researchers often resort to testing methods using *in silico* simulated data where the ground truth is presumably known (e.g., Bullmore et al., 2001; Calhoun et al., 2002; Roebroeck et al., 2005; Smith et al., 2011a). While the types of simulated data used by researchers are often simplistic (though see Seth et al., 2013), simulations with known ground truth remain necessary to test the validity and effectiveness of connectivity methods.

Recently, an analysis of the accuracy of several disparate connectivity methods was conducted using simulated data (Smith et al., 2011a). Dynamic causal modeling (Friston et al., 2003; DCM) was used, in reverse, to generate simulated fMRI data from known patterns of interregional connectivity and known noisy inputs. The various methods were then applied to the simulated data and their ability to recover the known connectivity patterns was compared. A problem with this study as well as previous comparisons of multiple methods is that the ground truth used for evaluation considered only the location and magnitude of the simulated interregional interactions; the nature of the interactions contained in the simulated data and the nature of the interactions a method was intended to identify were not considered. Given the ambiguity of the concept of connectivity, it is not immediately clear that the connectivity simulated by one method can be reasonably used to comparatively evaluate multiple disparate connectivity methods. The true interregional dynamics of *in vivo* fMRI data are surely complex and different connectivity measures may identify different aspects of these dynamics. In contrast, simulated data is simplistic by design, containing only the type of interregional interactions that were induced by the researcher. If the simulated data does not contain the type of interaction that a method was developed to identify, it is trivial to show that the method fails to identify the simulated connectivity. What is critical for method evaluation then is to simulate connectivity according to the type intended to be identified by the methods scrutinized. Therefore, only methods with compatible mathematical models of connectivity can be directly compared using the same simulated data.

The current study compares the accuracy of three different but fundamentally related methods, stochastic dynamic causal modeling for fMRI (sDCMf), switching linear dynamic systems for fMRI (sLDSf), and multivariate autoregressive models (MAR) using simulated data. First we present a unifying review of the foundations of several common connectivity methods. The methods are all placed into a similar multi-equation format that separates equations defining connectivity from those defining data observation. The underlying connectivity equation behind the three methods of interest is derived and a single equation is found to be applicable to all three. The form of the output equations for each method is defined and how these output equations may affect simulation accuracy is discussed. For completeness, parameter identification for each method is also briefly discussed. Finally, fMRI data from interconnected regions is simulated using the common equation and each method applied to attempt to recover the known connectivity. We conclude with a discussion of the strengths and failings of each method and suggest future directions for connectivity modeling.

## Unifying connectivity methods

First, a unifying discussion of common connectivity methods is presented to formally define what is meant by connectivity at the level of explicit equations. The discussion begins with the relatively simple equations behind so called “functional” connectivity measures. These simple equations are then expanded to form the basis of so called effective connectivity measures. The same equations are expanded again to produce the dynamic models of interest here. For clarity, constants in the equations necessary for centering or scaling have been omitted from the discussion unless necessary.

Assume a researcher is interested in approximating the interactions between signals arising from a set of brain regions of interest as measured by fMRI. Let *x*^*i*^_*t*_ be a scalar variable representing the value of the signal of interest at spatial location *i* and temporal location *t* and let *y*^*i*^_*t*_ be its measurement. To keep the discussion general, the specific nature of the signal of interest (e.g., local field potential or spatio-temporally integrated synaptic activity) will not be specified until later. Assume there are *n* spatial locations (brain regions) from which the signals of interest are measured and for which the connectivity is to be computed. Let *x*_*t*_ (resp. *y*_*t*_) be a column vector in ℜ^*n*^ containing the signals (resp. measurements) from regions [1 : *n*] at time *t* and **X** (resp. **Y**) be an *n* by *T* matrix collecting *x*_*t*_ (resp. *y*_*t*_) for a set of temporally contiguous time points [1τ, 2τ, 3τ … *T*τ] equally spaced by some distance τ in time.

## Relating observations to signals

The first step in generating any model is to state an observation equation that relates the signals of interest **X** to the observed data **Y**. A typical assumption is that the measurements are some direct function of the signals but corrupted by some noise due to the act of measurement and other unknown factors. A simple, analytically tractable form of this relation would be a linear function with Gaussian errors^1^ as shown in Equation 1.

(1) $$y_{t}=Cx_{t}+\xi_{t},\;\xi \sim \mathbb{N}(0,\;R)$$

Here ξ represents the corrupting noise with mean 0 and covariance **R**. The observation matrix **C** describes the extent to which the underlying signal at each spatial location is present at each measurement location. For some neuro-imaging methods where each measurement represents multiple underlying sources such as MEG, the matrix C could represent a (simplistic) model of source mixing and bone/scalp conduction. In fMRI, however, the observations are spatially specific. This spatial localization requires that x^i^ not influence y^j^ for i ≠ j; meaning **C** must be diagonal. The simplest means to achieve this for the moment is to set **C** to an identity matrix that can be ignored as in Equation 2. This simplified linear model will be expanded later in the discussion.

(2) $$y_{t}=x_{t}+\xi_{t},\;\xi \sim \mathbb{N}(0,\;R)$$

## Defining static functional connectivity

Different equations for the interactions among the signals yield different forms of connectivity. It is only by comparing and contrasting these equations that similarities and differences between various connectivity measures are realized. If a researcher assumes that the signals are independent through time (i.e., there is no function relating the signals at one time point to the signals at any other time point), they may choose from a family of so called static connectivity methods. The signals themselves are not known *a priori* and must be estimated from the data. Thus, the signals themselves are defined in terms of random variables. Assuming these variables are normally distributed and that the interactions among regions are linear results in the model shown in Equation 3.

(3) $$x_{t}=G\varepsilon_{t},\;\varepsilon \sim \mathbb{N}(0,\;Q)$$

Here **G** contains weights for mixing the as-yet-unknown, non-localized components ε together to produce the localized signals of interest. Given Equations 2 and 3, an explicit form for the distributions of X and Y can be specified as in 4.

(4) $$\begin{array}{l} X\sim \mathbb{N}(0,\;GQG^{T})\\ \;Y\sim \mathbb{N}(0,\;GQG^{T}+R) \end{array}$$

Obviously constraints must be placed on **G**, **Q**, and **R** for the signal **X** to be meaningful and for the model to be identified. A degenerate model (from the viewpoint of connectivity) could be obtained by simply setting **R** the covariance matrix of the measurements **Y** and setting **G** to a zero matrix, resulting in a rather useless signal. Without loss of generality, **Q** can be set to an identity matrix with the additional information subsumed by **G**. This simplification makes the components ε orthonormal (independent with unit variance). If the observation noise covariance, **R**, is restricted to an identity matrix multiplied by an arbitrary scalar, σ, and the limit taken as σ goes to zero (i.e., zero observation noise), Equations 2, 3, 4 form the Karhunen–Loève transform from signal processing also known as Principal Components Analysis (PCA; Jolliffe, 1986; Diamantaras and Kung, 1996; Tipping and Bishop, 1999)^2^. When PCA is used in (functional) connectivity analysis, typically only a few components with the greatest affect on the observed data variance are examined. Commonly used heuristics for selecting the number of components (e.g., 80 or 90% of the data variance; Jolliffe, 1986) are essentially assuming a non-zero bound for σ. If **R** is merely restricted to an arbitrary diagonal matrix, Equations 2, 3, and 4 describe the exploratory factor analysis (FA) method. In either case, the signals can be identified by maximizing the posterior probability of x given y as in 5 with the appropriate constraints on **R** (Roweis and Ghahramani, 1999). Note that as **R** approaches zero the covariance

(5) $$p(x|y)\sim \mathbb{N}\left(G^{T}{\left(GG^{T}+R\right)}^{-1}y,I-G^{T}{\left(GG^{T}+R\right)}^{-1}G\right)$$

of *p*(*x*|*y*) goes to zero and the maximum likelihood solution for **X** and **G** are obtainable via iterative least squares (Wold et al., 1987; Diamantaras and Kung, 1996; Wold, 1996).

The normality assumption for the signals and measurements can be relaxed by adding a non-linear function to Equation 3 as given in Equation 6.

(6) $$x_{t}=Gg(\varepsilon_{t}),\varepsilon \sim \mathbb{N}(0,I)$$

If **R** is again restricted to an identity matrix multiplied by an arbitrary scalar, σ, and the limit taken as σ goes to zero, Equations 2 and 6 describe the popular Independent Components Analysis (ICA) model^3^. If some if the components are assumed to be “noise” their variance is returned to **R** after identification of the zero noise system as in the PCA case above. It has been shown (Roweis and Ghahramani, 1999) that if the hyperbolic tangent function is used as the learning non-linearity in identifying the ICA demixing matrix, the non-linear function *g*(ε) in Equation 6 is given by Equation 7. Thus, ICA can be seen as both

(7) $$g(\varepsilon )=\ln\left(\tan\left(\frac{\pi }{4}\left(1+erf\left(\frac{\varepsilon }{\sqrt{2}}\right)\right)\right)\right)$$

a linear model with non-gaussian error or a non-linear model with Gaussian errors (Roweis and Ghahramani, 1999).

## Defining static effective connectivity

The connectivity contained within the **G** matrix of the PCA, FA, and ICA methods presented above are often referred to as “functional.” The signals *x*^*i*^ and *x*^*j*^ are functionally connected if the elements of the matrix **G** at *G*^(*i*, *k*)^ and *G*^(*j*, *k*)^ are both large in magnitude for at least one *k* ∈ [1 : *n*]. This would indicate the existence of a common component to the two signals of interest and therefore a non-zero correlation between them. There is, however, no inherent interaction, directionality, or “causality” between the signals in Equations 3 or 6 in their generic form. The signals *x*_*t*_ are functions of the componentsε_*t*_, not of each other.

**Figure 1:**
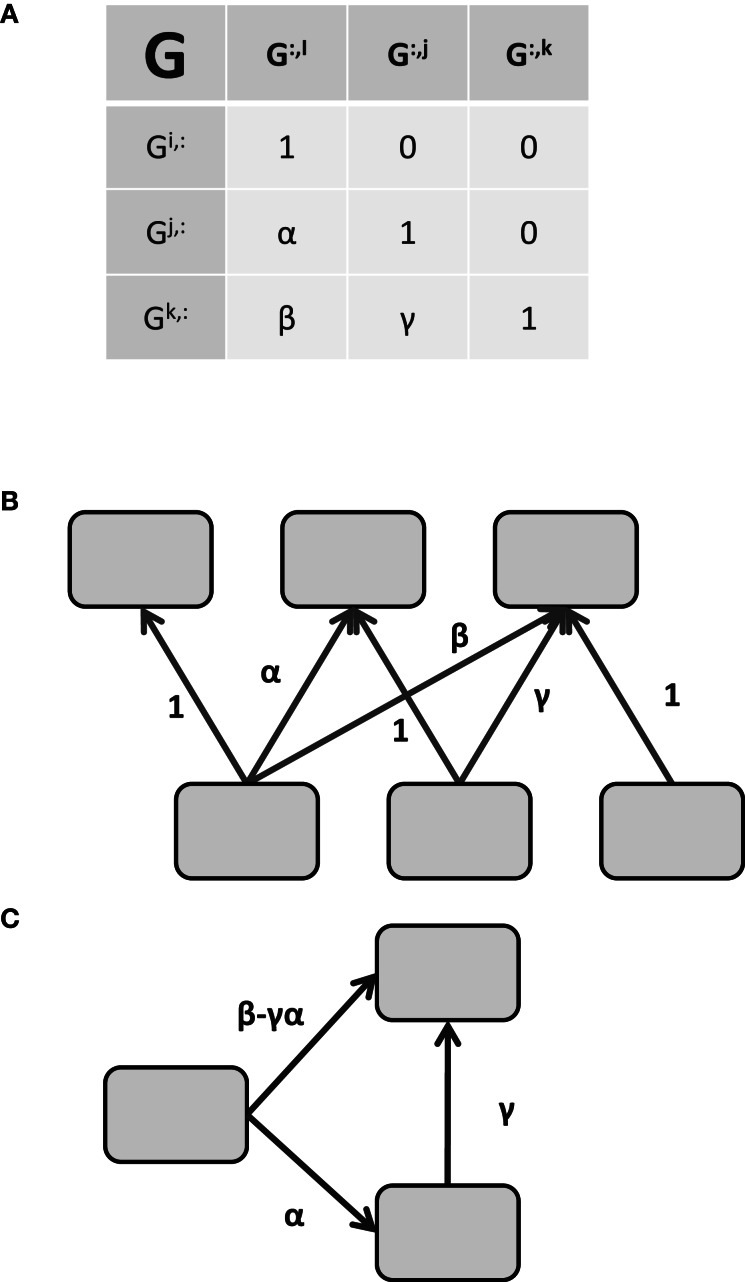
**Identifying effective connectivity from restricted functional connectivity. (A)** The G matrix relating unknown noise to the unobserved “neural” signals. Using the connectivity model of 1.6, the matrix G determines the covariance of the signal space via GG^T^. Connectivity in G is directional in the sense that G^*i*^ influences G^*k*^ via β but G^*k*^ does not influence G^*i*^. **(B)** Graphical depiction of the relationship between the common factors ε and the signals x implied by the pattern of values in **(A)**. The variable *x*^*i*^ is only influenced by ε^*i*^ while *x*^*j*^ is influenced by ε^*i*^ and ε^*j*^, and so on. **(C)** Effective connectivity between the signals. The effective connectivity depicted in C is a direct consequence of the matrix G in **(A)**. See the text for the solution.

Effective connectivity is typically taken to imply causality or at least directionality to the connectivity (Valdés-Sosa et al., 2011). Returning to the linear model of Equation 3 with **Q** = **I**, if **R** is again restricted to a diagonal matrix, and **G** is constrained *a priori* to have at most *n*(*n* − 1)/2 non-zero values and be acyclic (satisfied if **G** is lower-triangular), Equations 2,3, and 4 describe the confirmatory FA model, also known as Structural Equation Modeling (SEM)^4^. With appropriate priors, 2 through 4 can also describe Linear Gaussian Bayesian Networks (BN, Rajapakse and Zhou, 2007). To see how the lower triangular (or other acyclic) restriction implies directionality in Equation 3, consider the three element model shown in Figure 1. The matrix **G** is shown in Figure 1A implying the functional connectivity (i.e., common components) shown in Figure 1B. Ignoring terms with zero coefficients, the restrictions described above result in the following set of equations for the **G** matrix of Figure 1A.

(8) $$\begin{array}{l} x_{t}^{i}=\varepsilon_{t}^{i}\\ x_{t}^{j}=\alpha \varepsilon_{t}^{i}+\varepsilon_{t}^{j}\\ x_{t}^{k}=\beta \varepsilon_{t}^{i}+\gamma \varepsilon_{t}^{j}+\varepsilon_{t}^{k} \end{array}$$

Since the signal *x*^*i*^ is localized in space and *x*^*i*^ and ε^*i*^ are equivalent, the component ε^*i*^ is localized as well. Thus, we are justified in rewriting the signal in region *j*, *x*^*j*^_*t*_, as a function of the region *x*^*i*^_*t*_ and its residual error ε^*j*^_*t*_ with α indicating the strength of the connectivity from region *i* to region *j*.

(9) $$x_{t}^{j}=\alpha x_{t}^{i}+\varepsilon_{t}^{\;j}$$

Similarly, *x*^*k*^_*t*_ may be rewritten as a function of *x*^*i*^_*t*_ and *x*^*j*^_*t*_.

(10) $$\begin{array}{l} x_{t}^{k}=\beta x_{t}^{i}+\gamma \left(x_{t}^{j}-\alpha x_{t}^{i}\right)+\varepsilon_{t}^{k}\\ x_{t}^{k}=\left(\beta -\gamma \alpha \right)x_{t}^{i}+\gamma x_{t}^{j}+\varepsilon_{t}^{k} \end{array}$$

Therefore, the directional connectivity graph in Figure 1C is a direct result of the Equations in 8. Returning to the non-linear model in Equation 6 and again restricting **G** to be lower-triangular, the functional connectivity of the ICA method becomes the effective connectivity of the Linear Non-Gaussian Acyclic method (LiNGAM, Shimizu et al., 2006) in a manner directly analogous to the relation between exploratory FA and SEM. Thus, the type of connectivity contained in the static effective connectivity methods SEM and LiNGAM is not inherently different from their static functional connectivity counterparts as they rely on the same underlying equations; they are merely restricted subsets of the same connectivity model. In light of this similarity, and to keep consonance with terminology from other fields, we suggest the use of the term “confirmatory connectivity” and “exploratory connectivity” in place of effective and functional respectively for these static methods.

## Defining dynamic connectivity in discrete time

SEM, BN, and LiNGAM are static connectivity methods in that they require the assumption of no temporal structure to the data (though see e.g., Fisher, 1970 for a dynamic interpretation of SEM). That is, any permutation of the temporal ordering of **Y** will produce identical values in **G** for these methods. Though a useful fiction, this assumption is untenable when the observations are closely spaced in time as is the case for fMRI. If a linear temporal dependency of *x*_*t*_ on its past values (e.g., *x*_*t* − τ_) is included in Equation 3, the multivariate autoregressive (MAR^5^) model is recovered.

(11) $$x_{t}=Ax_{t\;-\tau }+G\varepsilon_{t},\varepsilon \sim \mathbb{N}(0,I)$$

Note that the measurement function given by Equation 2 is still being used with Equation 11 for the MAR model^6^. Note as well that there are now two “connectivity” matrices. As before, **G** describes the connectivity that is instantaneous relative to the sampling rate, or occurring “within” the sample interval. This connectivity can be thought of as residual static exploratory (functional) connectivity or, if restricted as in SEM, residual static confirmatory (effective) connectivity. In addition, the new matrix **A** describes connectivity across time. The connectivity in **A** is directional and causal. However, if **G** is not strictly diagonal, the elements of **A** as identified in 11 must be interpreted with some caution as they do not account for all the interregional interactions (Sims, 1980; Sims and Zha, 1998; Lütkepohl, 2007; Rubio-Ramírez et al., 2010; Chen et al., 2011; Smith et al., 2011b). To identify the unique effect of an externally induced change in one variable on the system described by 11 requires an additional restrictions on the within time connectivity **G** as seen before with SEM (Rubio-Ramírez et al., 2010). If **G** is invertable structured such that it can be written in lower-triangular form by reordering the variables, the within time connectivity, **S_0_**, is given by **G**^−1^ while the cross time connectivity, **S_1_**, is given by **AG**^−1^ (Sims, 1980; Rubio-Ramírez et al., 2010). While most MAR modeling for fMRI has been conducted using the framework of Granger Causality^7^ and has considered only the through time connectivity in the **A** matrix (cf. Seth, 2005) analysis of both **A** and **G** may be more useful to better understand connectivity at the relatively low sampling rate of fMRI (Chen et al., 2011; Smith et al., 2011b).

For completeness Equation 12 gives a generic form of the MAR connectivity equation that includes external inputs, *v*_*t*_, and possible temporal variability for the connectivity and noise covariance. The *t* subscript on the matrices indicates that these values may change with time

(12) $$x_{t}=A_{t}x_{t-\tau }+B_{t}v_{t}+G_{t}\varepsilon_{t},\varepsilon \sim \mathbb{N}(0,I)$$

(e.g., with changing task condition). This generic connectivity equation contains stationary SEM (**A**_*t*_ = 0, **B**_*t*_ = **B**, **G**_*t*_ = **G**) and stationary MAR (**A**_*t*_ = **A**, **B**_*t*_ = **B**, **G**_*t*_ = **G**) as special limited cases. Obviously, the MAR connectivity equation should not be used to simulate data for SEM with a non-zero **A** matrix (see Fisher, 1970 for a possible exception to this rule).

The full temporal variability in 12 is difficult to solve; identifying a unique full **A** matrix for each time-point is a severely under-determined problem. Methods for identifying **X** and each unique **A**_*t*_ do exist; for example by assuming a model for **A**_*t*_ given **A**_*t* − τ_ (Wan and van der Merwe, 2001; Havlicek et al., 2011; Smith et al., 2011b). However, typically **A**_*t*_ is more constrained. If the matrices in 12 are assumed constant over some interval and interval boundaries are known, standard methods for identifying MAR model parameters may be used within the intervals. Bilinear models, which assume **A**_*t*_ is a function of a constant matrix **A** altered by a known external variable are another simple method to identify a constrained **A**_*t*_ (cf. Penny et al., 2005; Makni et al., 2007; Ryali et al., 2011). The bilinear model is easily presented in the form of 12 by setting *A*_*t*_ to *A* + ∑_*j*_*H*_*j*_*u*^*j*^_*t*_ where **H** is the (non-temporally varying) bilinear term and *u*_*t*_ is the known input influencing this term (Smith et al., 2010)^8^. If *u*_*t*_ is simply an indexing variable, the result is similar to the constant interval method. However, the bilinear model does not account for non-stationarity in the error and other inputs, holding **G**_*t*_ and **B**_*t*_ to constant matrices. If a small number of distinct **A**, **B,** and **G** matrices are assumed to exist (e.g., *A*^*i*^, *B*^*i*^, *G*^*i*^, *i* ∈ [1, 2, …, *p*]) and the probability of their application at a time *t* is conditioned on an additional variable *s* with the same temporal index (e.g., *p*(*A*_*t*_ = *A*^*i*^ | *s*_*t*_ = *s*^*i*^)), the switching linear dynamic system (sLDSf) model is recovered (Smith et al., 2010). If the index variable *s* is known, the parameters of the sLDSf model can be recovered in a similar manner as in the constant interval case. If the index variable s is unknown, the problem is more difficult, but solvable (Kim, 1994; Murphy, 1998; Barber, 2006; Fox et al., 2010). The connectivity of sLDSf can be as general as Equation 12 assuming the limited set of **A**^*i*^, **B**^*i*^, and **G**^*i*^ matrices in the sLDSf model form a basis spanning the space of possible **A**_*t*_, **B**_*t*_, and **G**_*t*_ matrices such that any A_*t*_ matrix (resp. **B**_*t*_, **G**_*t*_) can be represented as a weighted combination of the given **A**^*i*^ (resp. **B**^*i*^, **G**^*i*^) matrices (Smith et al., 2011b).

## Defining dynamic connectivity in continuous time

The MAR and sLDSf methods are defined in discrete time. That is, the connectivity equations describe the evolution of values of the signals *x*_*t*_ over a fixed set of time-points *t* ∈ [1 τ 2 τ 3 τ …]. These equations can be transformed, without error, such that they describe the differences between temporally contiguous values of the signals *x*_*t*_ (i.e., *x*_*n*τ_ – *x*_(*n* − 1)τ_). This is the so called “delta” form of the MAR model and is shown in Equation 13.

(13) $$(x_{t\;+\tau }-x_{t})/\tau ={\bar{A}}_{t}x_{t}+{\bar{B}}_{t}v_{t}+{\bar{G}}_{t}\varepsilon_{t}$$

For the technically minded, the conversion from 12 to 13 is given in **Appendix B**. The left-hand side of Equation 13 can be seen as an Euler approximation to a derivative, thus as the sampling rate τ approaches 0, Equation 13 approaches the derivative of *x*_*t*_ as in 14.

(14) $${\dot{x}}_{t}={\tilde{A}}_{t}x_{t}+{\tilde{B}}_{t}v_{t}+{\dot{\eta }}_{t}$$

Because it is expressed in terms of a derivative, if the value of *x* is known at some time point (e.g., *x*_0_), 14 can be used to identify a value of *x*_*t*_ for any value of *t* > 0. Thus, 14 is referred to as continuous time. The continuous time matrices in 14 have been marked with the tilde to distinguish them from the discrete time versions in 12. The vector ${\dot{\eta }}_{t}$ is a white noise disturbance which must technically have infinite variance and its full treatment is beyond the scope of the current discussion. Because of this, Equation 14 is more often expressed via a stochastic integral of the integrated version of ${\dot{\eta }}_{t}$ as

(15) $$dx={\tilde{A}}_{t}x_{t}dt+{\tilde{B}}_{t}v_{t}dt+d\eta_{t},\text{E}\left[\left[d\eta \right]\right]\text{E}{\left[\left[d\eta \right]\right]}^{T}=\tilde{Q}dt$$

in 15 where E[*x*] indicates the expected value of *x* and $\tilde{Q}dt$ can be considered an incremental covariance matrix. If the noise process in 14 is removed, the result is a deterministic differential equation similar to that used to describe connectivity in the original deterministic Dynamic Causal Model (dDCM, Friston et al., 2003). All that is needed to recover dDCM from 14 with ${\dot{\eta }}_{t}=0$ is to replace${\tilde{A}}_{t}$ with $\tilde{A}+\sum_{j}{\tilde{H}}_{j}u_{t}^{j}$ to form the bilinear model as done in the previous section. The bilinear continuous time neural connectivity of sDCMf can be recovered from 15 in a similar manner and is given in Equation 16.

(16) $$\begin{array}{l} dx=\left(\tilde{A}+\sum_{j}{\tilde{H}}_{j}u_{t}^{j}\right)x_{t}dt+\tilde{B}v_{t}dt+d\eta_{t},\text{E}\left[\left[d\eta \right]\right]\text{E}{\left[\left[d\eta \right]\right]}^{T}\\ \;=\tilde{Q}dt \end{array}$$

The letters used as matrix labels in 16 have been changed from those of Friston et al. (2003) to remain consistent with the equations above.

The relation between continuous time connectivity expressed in 16 and the discrete time connectivity of 12 has been well studied (cf. Åström, 1970; Bar-Shalom et al., 2001; Ljung and Wills, 2010). Assuming the system matrices ($\tilde{A}$, $\tilde{B}$, and $\tilde{Q}$) and the input *v*_*t*_ are constant over the interval [*t* − τ : *t*] then the exact system matrices for an equivalent discrete time system can be derived from the continuous time versions as follows:

(17) $$\begin{array}{l} A_{t}=e^{\tau {\tilde{A}}_{t}}\\ B_{t}={\tilde{A}}_{t}^{-1}\left(e^{\tau {\tilde{A}}_{t}}-I\right){\tilde{B}}_{t} \end{array}$$

where *e*^*A*^ indicates the matrix exponential of **A** and **A**^−1^ indicates the inverse of **A**. The matrix **B**_*t*_ can still be computed from ${\tilde{B}}_{t}$ even if ${\tilde{A}}_{t}$ is singular. The covariance of the stochastic input for the discrete time system can also be derived from the stochastic continuous time system via the Equations in 18.

(18) $$\begin{array}{l} \varepsilon_{t}=\int_{s=t-\tau }^{s=\;t}e^{{\tilde{A}}_{t-\tau +s}}d\eta_{s}\\ Q=\int_{s=0}^{s=\tau }e^{{\tilde{A}}_{s}}\tilde{Q}e^{{\tilde{A}}_{s}^{T}}ds \end{array}$$

Thus, the corresponding discrete time **Q** is the solution to the continuous Lyapunov equation:

(19) $${\tilde{A}}_{t}Q+Q{\tilde{A}}_{t}^{T}+\tilde{Q}-e^{\tau {\tilde{A}}_{t}}\tilde{Q}e^{\tau {\tilde{A}}_{t}^{T}}=0$$

To reduce this **Q** to an identity matrix as assumed in 12 and recover **G**, recall that if a matrix **W** is the Cholesky factor of a symmetric matrix **Z** then:

(20) $$\begin{array}{l} W=chol(Z)\\ Z=WIW^{T} \end{array}$$

Examining Equations 3 and 4 we see that if the covariance of ε_*t*_ is **Q**, then the covariance of *G*ε_*t*_ is *GQG*^*T*^. Thus, the matrix **G** from 12, assuming identity covariance of ε, is derived from the matrix $\tilde{Q}$ in 15 by solving for **Q** in 19 and then taking the Cholesky factor of the resulting matrix^9^.

It is important to stress that the mapping from continuous time to discrete time is subjective. That is, assuming an appropriate sampling interval, there is one and only one discrete time system that corresponds to a given continuous time system. The matrix exponential function in Equation 17 is unique as is the solution to 19^10^. Assuming the continuous time matrices contain no frequencies higher than the half the sampling rate, the converse is also true. The inverse of the matrix exponential, the matrix log, may or may not have a unique solution (Culver, 1966), but other methods exist for discrete-to-continuous conversion (Raol et al., 1987; Åström and Wittenmark, 1996; Franklin et al., 1997). Unfortunately, if the sampling period is insufficient, there will be many continuous time systems that could correspond to a given discrete time system.

To summarize, Equation 15 and its equivalent discrete time counterpart in Equation 12, as derived by Equations 17 through 20, represent generic dynamic effective connectivity equations that contain dDCM, sDCMf, sLDSf, MAR, SEM, FA, and PCA as special cases. However, not all of the special cases are compatible. Assuming appropriate care is taken with scaling, definition, and conversion of the system matrices, sDCMf, sLDSf, and MAR can all share the single connectivity Equation 12. However, dDCM is not compatible with sLDSf, MAR or any of the static connectivity measures. The dDCM form of 15 assumes ${\tilde{Q}}_{t}$ (and thus *G*_*t*_) is zero and all connectivity is described by $\tilde{A}+\sum_{j}{\tilde{H}}_{j}u_{t}^{j}$ while the static connectivity measures such as SEM assume *A*_*t*_ (and thus (${\tilde{A}}_{t}$) is zero and all connectivity is described by *G*_*t*_. Therefore, using dDCM to generate simulated fMRI data for testing static connectivity methods (e.g., SEM, FA, PCA, ICA, correlations, partial correlations, etc.), because of its lack of compatibility with these measures, is inappropriate.

## Diverging observation models

The discussion above focused on identifying the connectivity equations; the observation equation was ignored beyond the assumption that *y*^*i*^ was dependent only on *x*^*i*^ and not *x*^*j*^ for *i* ≠ *j* and assuming various restrictions on the observation noise covariance **R**. Though 12 represent the common connectivity equation for sDCMf, sLDSf, and MAR, the methods do not share common observation equations. The observation equations for each of the three methods are discussed below in turn.

### MAR

The MAR observation equation is the simplest of the three methods discussed here. The observation equation assumed by a MAR analysis of fMRI data is essentially Equation 2. However, 2 can be further simplified in the MAR case by setting ξ and **R** to 0 and placing any observation noise variance into the variance of ε in Equation 12 and scaling the system matrices accordingly. This would make **Q** an arbitrary diagonal matrix. It could be argued that this simplistic observation equation is a poor model for mapping from predominantly neurally based variables (e.g., local field potentials) to the hemodynamic variable observed via fMRI (Smith et al., 2011a). The BOLD signal obtained in fMRI is an epiphenomenon arising from neural, glial, and vascular sources (Logothetis et al., 2001; Logothetis and Pfeuffer, 2004; Logothetis, 2008). It has been assumed that BOLD signals in one region do not causally influence BOLD signals in other regions. This would seem to invalidate the MAR observation equation since the signals, **X**, would be defined and interact in “hemodynamic” space. However, the BOLD response can be accurately modeled as a causal low pass filter (Henson, 2003). While the exact shape of this filter may vary from subject to subject and from region to region within subjects, there is a common generic shape of the BOLD filter (Aguirre et al., 1998; Handwerker et al., 2004, 2012). The impulse response of this filter can be approximated by a Poisson function or mixture of two Beta functions (Friston et al., 1994). To the extent that the transfer function of the system described by Equation 12 contains no frequencies higher than those passed by the BOLD filter, passage of **X** through this filter would result in a relatively undistorted temporal shift of the true signal (Barnet and Seth, 2011; Seth et al., 2013). Unfortunately, a change from one set of system matrices to another in the connectivity equation would likely induce a high frequency component in **X**. This suggests that MAR is likely better suited to the so-called resting state fMRI or slow block design experiments than to event related or other rapidly changing designs. However, the output equation of MAR requires no additional parameters to be estimated and the MAR model can be identified in closed form.

### sLDSF

The observation equations used in sLDSf are based on the linear convolution model of Penny et al. (2005). The sLDSf observation equations are presented in Equations 21 and 22.

(21) $$y_{t}=\beta \phi z_{t}+Dv_{t}+\zeta_{t},\zeta \sim \mathbb{N}(0,R)$$

(22) $$z_{t}=\left[x_{t},x_{t-\tau },x_{t-2\tau },x_{t-3\tau },\ldots ,x_{t\;-(h-1)\tau }\right]$$

The variable *z*_*t*_ contains *h* errorless lagged copies of the signals *x* from *x*_*t* − (*h* − 1)τ_ to *x*_*t*_. The observation, *y*_*t*_, is an instantaneous linear function of *z*_*t*_ any additional observation level input *v*_*t*_ (e.g., movement related variables) and noise ζ_*t*_ with a diagonal covariance matrix **R**^*ij*^ = 0 for *i* ≠ *j*. The matrix Φ is an *a priori* known set of basis vectors that span the likely variability in the hemodynamic impulse response function (hIRF) such as a canonical hemodynamic response and its derivatives with respect to time and dispersion (Penny et al., 2005). The matrix β contains regionally specific weights for these bases to generate a unique hIRF β^*i*^Φ. The linear output β^*i*^Φ*Z*^*i*^_*t*_ is thus equivalent to convolving each signal with a regionally specific hemodynamic response (Smith et al., 2010). The SLDSf output equations with three basis vectors require the estimation of three additional parameters per region.

### sDCMF

The output equations for sDCMf are the most complex of the three methods. DCMf uses a non-linear dynamic system to model the biophysical states that engender the BOLD signal (Friston et al., 2003). The DCMf output equations embody the Balloon–Windkessel model (Buxton et al., 1998; Mandeville et al., 1999; Friston et al., 2003; Riera et al., 2004; Buxton, 2012). The regionally localized signal xi causes an increase in an equally localized vasodilatory signal. Inflow responds to this signal and affects blood volume as well as feeding back to the vasodilatory signal. Change in deoxyhemoglobin content is a function of flow, volume, oxygen extraction as well as its past. This complex interaction can be summarized with Equations 23 through 26 where s is the vasodilatory signal, *f* is inflow, *v* is blood volume, *q* is:

(23) $$\frac{ds}{dt}=x-\kappa s-\gamma (f-1)$$

(24) $$\frac{df}{dt}=s$$

(25) $$\tau \frac{dv}{dt}=f-v^{1/\alpha }$$

(26) $$\tau \frac{dq}{dt}=f(1-{\left(1-\rho \right)}^{1/f}/\rho -v^{1/\alpha }/v$$

deoxyhemoglobin content, alpha is Grubb's exponent and rho is resting oxygen extraction fraction. The observed BOLD signal is then taken to be a static non-linear function of volume and deoxyhemoglobin with additional biophysical parameters as in Equation 27. In all, the output

(27) $$y=0.02(9\rho -\rho^{2}+1.8-2q/v-2\rho v-0.2v)$$

equations of sDCMf require five additional parameters to be estimated per region.

## Parameter estimation

The parameter estimation methods of MAR, SLDSf, and sDCMf are briefly reviewed. For more detailed descriptions see (Murphy, 1998; Neumaier and Schneider, 2001; Lütkepohl, 2007; Daunizeau et al., 2009; Friston et al., 2010; Li et al., 2011; Smith et al., 2010).

### MAR

Coefficients of MAR models can be obtained through linear regression of past on future values using least-squares methods (Lütkepohl, 2007). To avoid numerical issues related to forming and inverting the covariance of the predictor matrix, we implemented the regression via QR factorization (Stewart, 1987). For each simulation, all time points within a condition were selected and the first four observations per block were discarded. Inputs were shifted forward in time by 4 s to accommodate the delay in the peak hemodynamic response^11^. The inputs and observations were combined to form a single design matrix and used to predict the observations 1 s after the times included.

### sLDSF

The parameters of the sLDSf model are identified using an iterative Bayesian Expectation Maximization (EM, Dempster et al., 1977) algorithm written by the first author in a combination of Matlab and ANSI C. In brief, starting estimates of the system matrices based on a MAR model are used to estimate the signals given the observed data using Kalman filtering/smoothing (Bar-Shalom et al., 2001; Haykin, 2002; Lütkepohl, 2007; Smith et al., 2010). The likelihood of the data given the model is also calculated. This is the expectation step of the EM algorithm. The likelihood is maximized given these signals by updating the system matrices. The matrices are updated by setting the derivatives of the likelihood function with respect to each of the system matrices to zero and solving (Shumway and Stoffer, 1982; Ghahramani and Hinton, 1996; Murphy, 1998; Penny et al., 2005). These updated system matrices are then used to estimate new signals. This iterative process is repeated until the change in likelihood at each step drops below a specified threshold.

The likelihood of the data given the model was modified to include priors on the system matrices (cf. Makni et al., 2007; Ryali et al., 2011). The parameter identification method implemented here used the Empirical Bayes framework (Bishop, 2006). A multivariate normal-inverse gamma prior distribution was used for β and the diagonal elements of **R** (Bishop, 2006). The prior mean of β was set to [1,0,0] at each region for the canonical hIRF, its temporal derivative, and its dispersion derivative, respectively. The necessary hyperparameters were estimated from the data (Bishop, 2006). A matrix-normal-Wishart density was used for the conjugate prior for **A**, **B**, and **Q** given **X** and **u** (Minka, 2000) and shown in 28. The prior mean of **A** was set to the

(28) $$p\left([A,B]|\left[\begin{array}{c} X\\ u \end{array}\right]\right)\sim \mathbb{N}W^{-1}\times \left(\left[\frac{0.5}{TR}I,0\right],\sum_{j}\alpha_{j}\left[\begin{array}{c} X\\ u \end{array}\right]S_{j}{\left[\begin{array}{c} X\\ u \end{array}\right]}^{T},zI,z\right)$$

identity matrix multiplied by 0.5/TR and the prior mean of **B** was set to zero. Two separate variances were estimated for the values in **A** and **B**; one for the elements of **A** and one for the elements of **B**. The matrix **S**j in 28 is an appropriate selection matrix to allow for the separate variances and z is the estimated degrees of freedom. The necessary hyperparameters were estimated from the data (Minka, 2000).

### sDCMF

Parameter identification for sDCMf was performed with SPM12 (http://www.fil.ion.ucl.ac.uk/spm 4750 2012-05-24 14:05:41Z). This version uses the generalized filtering method described by Friston et al. (2010) and Li et al. (2011). Parameter identification involves approximating the conditional density on the unknown parameters and signals and maximizing an approximate bound on the log evidence using a variational Bayesian method. The function spm_dcm_estimate included in SPM12 was called via a separate script with the following parameter settings: stochastic = 1; center = 1; endogenous = 0; delays = 0; *dt* = 1 s. In addition, if for a given simulation any element of the simulated A matrix was zero for all conditions in that simulation, sDCMf, was informed to set this element to zero.

### Implications

The commonality of the connectivity equations for sDCMf, SLDSf, and MAR suggest the following steps to simulate *in silico* interregional effective connectivity for these methods. First, the continuous time matrices ${\tilde{A}}_{t}{\tilde{B}}_{t}{\tilde{Q}}_{t}$ and the vector *v*_*t*_ are defined in the simulation for all possible values of *t*. Second, a small step size, τ, is chosen such that $\overset{⌢}{A_{t}}{\tilde{B}}_{t}{\tilde{Q}}_{t}$ and *v*_*t*_ are all constant on the interval *t*–τ to *t*. The discrete time matrices *A*_*t*_, *B*_*t*_, and *Q*_*t*_ and/or *G*_*t*_ for step size τ are identified using 17 and solving 19. The signal data, X, is then simulated using these discrete time matrices at a step size τ with suitable random noise passed through G. We stress that this is not equivalent to integrating a deterministic version of 15 using the Euler or Runge–Kutta method with a temporal step of τ and then adding noise with covariance ${\tilde{Q}}_{t}$ after each step. The matrix ${\tilde{Q}}_{t}$ is not the appropriate noise covariance for such discrete time steps because the noise also needs to be integrated over this period (Ljung and Wills, 2010). Simulated fMRI data can then be generated using an appropriate function of the simulated signal that is consistent with real fMRI data but is not the actual output function of any of these methods. Parameters of sDCMf, sLDSf, and MAR models are identified for the simulated fMRI data. The true system matrices and the identified sDCMf system matrices are then converted, without error, to a step size common to the sLDSf and MAR (typically the simulated TR). Once all system matrices are expressed in a common format they can be directly compared.

To produce simulated fMRI data from the signal data created as above, one could apply method specific output equations to generate method specific data. However, because the MAR output equation does explicitly not model the BOLD response this would not be a realistic test of the utility of MAR for fMRI. To avoid bias, a different output model was chosen for the simulations. The mixture of two betas model implemented in the function spm_hrf() distributed with SPM8 () was used to create hIRFs that would then be convolved with the signals. The spm_hrf() function essentially forms two time series from beta probability distributions and subtracts the second from the first. The purpose of the second is to model the post-stimulus undershoot seen in BOLD data. The function has eight parameters: step time, delay of response peak from onset, delay of undershoot peak from onset, dispersion of response, dispersion of undershoot, ratio of response to undershoot, onset length, and length of kernel. To create variable hIRFs, three parameters to the spm_hrf() function were allowed to vary at random from typical values; delay of response peak from onset, delay of undershoot peak from onset, and ratio of response to undershoot.

The resulting variable hIRFs, while distinct from the exact sDCMf or sLDSf output equations, should be well modeled by both methods. Though DCMf can model non-linear aspects of the hemodynamic response, these non-linearities are only important when using an estimated hemodynamic response from a short stimulus to predict the response to a longer stimulus or when considering only overly simplistic models of the neural response (Yesilyurt et al., 2008; Marxen et al., 2012). The Balloon–Windkessel model as implemented in DCMf and the linear convolution model produce equivalent outputs when inputs are slow and no distinction is typically seen between them (Seth et al., 2013). To ensure both DCMf and SLDSf were able to mimic the simulation hIRFs, both the basis function method of SLDSf and the non-linear system method of DCMf were directly tested. To test the SLDSf basis method, fifty random hIRFs were generated and optimal weights on the three bases were identified using ordinary least squares. The mean *R*^2^ for the basis function method was 0.9966 and the minimum *R*^2^ was 0.9809. For the non-linear system method of DCMf, the best fitting set of parameters (in terms of squared error) for the hemodynamic model were identified for the same 50 random hIRFs using the Levenburg–Marquardt method as implemented in the Matlab function lsqnonlin(). Both the mean and minimum *r*^2^ for the non-linear system method were greater than 0.9999. While a paired *t*-test indicated that the superior performance of the non-linear system method over the basis function method was statistically significant [*t*_(49)_ = 5.96, *p* < 0.001] the basis function method fits were extraordinarily accurate and in practice the difference is likely meaningless.

### Methods

All simulations were performed in the Matlab (Mathworks Inc., Natic MA) 2012a environment. Matlab functions for generating the simulations are included as supplementary material. Briefly, for each individual simulation with *n* regions, an *n*-dimensional Hurwitz stable continuous time transition matrix was created for each of *p* conditions. For a continuous time transition matrix to be Hurwitz stable, all of its eignvalues must be in the open left half plane of the complex plane (i.e., negative real part). Stable transition matrices were created by drawing *n* real random values from a gamma distribution with shape parameter 3 and scale parameter 0.275 and negating them. These *n* negative values were treated as eigenvalues and left-multiplied, right-divided by an orthonormal *n* by *n* eigenvector matrix created using the randn() function and orthonormalized using the orth() function. The resulting symmetric matrix was rotated using an additional random *n* by *n* matrix. Entries in the resulting matrix with absolute value less than 0.1 were set to zero. The continued continuous time stability of the resulting matrix was tested by examining the eigenvalues of the constructed matrix. If a non-stable matrix was created, the process was repeated. The instantaneous error covariance matrix was created using *n* random positive eigenvalues from the range zero to one generated using the rand() function and a second orthonormal *n* by *n* eigenvector matrix without further rotation to maintain symmetry. The matrix describing the effect of external input on the state space was generated using the randn() function and all entries with absolute value less than 0.1 were set to zero. The resulting matrices were converted to their discrete time counterparts (A, B, and G) using Equations 14 to 17 with a step size of 25 ms. Discrete time versions of the true matrices were also created with a step size of 1000 ms for evaluation purposes.

The simulation consisted of a 10 min (600 s) time series having 25 blocks with 24 s per block. The ordering of the blocks in each simulation was created as follows. For simulations consisting of two conditions (i.e., *p* = 2), an ABAB design was used. For three or more conditions (i.e., *p* > 2) a Markov transition matrix was created with zero probability of remaining within condition an equal probability of jumping to any other condition. A random ordering of 25 blocks was generated using this Markov matrix and the rand() function.

Inputs to the state space were generated by randomly setting 60 of the 25 ms steps to 1 for each of two inputs. Discrete time noise was created from an orthonormal distribution for every 25 ms of a 600 s period. The signal data was then created at each 25 ms step using the condition series, input, noise, and system matrices according to Equation 11.

**Figure 2:**
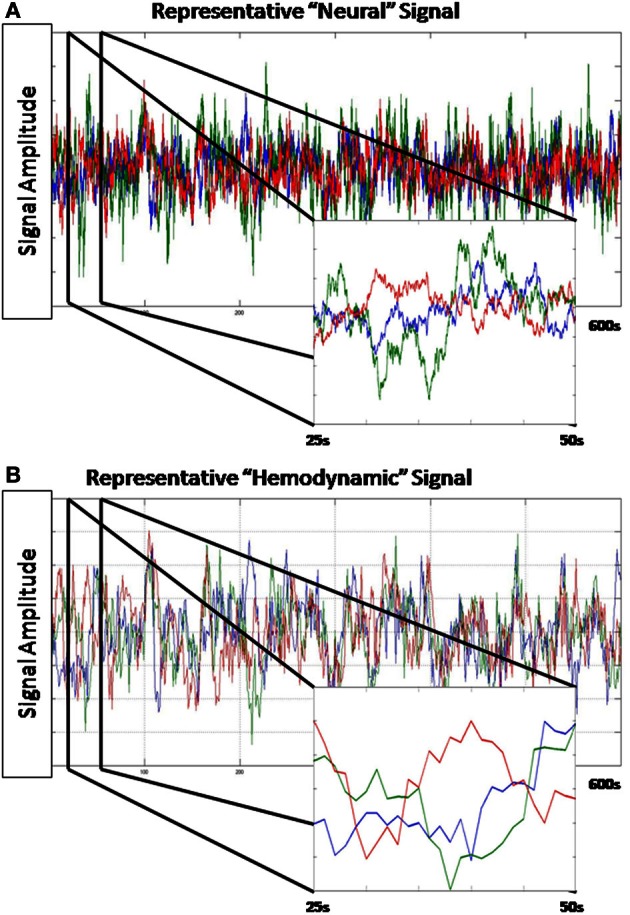
**Representative data from a simulation. (A)** Shown is 600 s of the “neural” level signal from a single simulation. **(A)** inset is a close-up of 25 s from this time series. This time series is sampled at 0.025 Hz. **(B)** The hemodynamic response generated by this “neural” signal. **(B)** inset is a close-up of the same 25 s segment. This time series is sampled at 1 Hz.

A hIRF was created for each of the n regions at the 25 ms step size. The mixture of two betas model implemented in the function spm_hrf() distributed with SPM8 (www.fil.ion.ucl.ac.uk/spm/) was used to create the hIRFs with the following parameter settings: *TR* = 0.025, delay of response peak from onset = 3.5 s + [0–3]s, delay of undershoot peak from onset = 12 s + [0–3]s, dispersion of response = 1, dispersion of undershoot = 1, ratio of response to undershoot = 11 + [0–3], length of kernel = 20 s. For each region, the signal from that region was convolved with the region's impulse response and then down-sampled by selecting every 40th value to create a 1 s step size. Orthogonal white noise was then added with a SNR of 6.0206 db. The resulting observations were mean centered and scaled to unit variance. A sample time series is presented in Figure 2.

For testing the methods, the number of regions ∈ {3, 5} and conditions ∈ {2, 3} in the simulated dataset were varied, and 50 different simulations were created for each combination of parameter settings. Each of the methods was then applied to the simulated data. Analysis was conducted on a custom workstation (AVADirect, www.avadirect.com, Cleveland, OH) with two Xeon X5690 processors and 48GiB RAM running RedHat Enterprise Linux Release 6.1, Kernel 2.6.32-131.13.1.el6.x86_64. Identification of MAR models was essentially instantaneous for all simulations. Identification of sLDSf models took approximately 36 s for the most complex simulations while identification of sDCMf models took approximately 6495 s for the same simulations. Because of the time cost for estimating the sDCMf models, more complex simulations (e.g., more than 5 regions or more than 3 conditions) were not examined.

To directly compare the three methods, the continuous time transition matrices identified by sDCMf were converted to their discrete time counterparts at a 1 s sampling rate as were the original true continuous time simulation matrices. The sLDSf and MAR transition matrices were identified at this sampling rate and thus did not require further manipulation. Four measures were chosen to assess the relative accuracy of the methods. First, the *r*^2^ for the comparison between the true and estimated A matrices each method on each simulation according to Equation 29.

(29) $$r^{2}=1-\sum_{\text{elements}}{\left(A^{\text{true}}-A^{est}\right)}^{2}/{\left(A^{\text{true}}-{\bar{A}}^{\text{true}}\right)}^{2}$$

Note that while 29 is the correct formula, because the result is a subtraction of a squared value from one, there is a possibility that the resulting *r*^2^ will be negative if the prediction of A is worse than simply guessing the mean of A. For sDCMf and sLDSf, the scaling of the signals of interest, and thus the A matrices, is arbitrary. Considering the sLDSf method equations, given an arbitrary invertible matrix T of the correct dimensions, a model with system matrices A and C is equivalent to one with matrices AT and CT^−1^. Thus, any scaling that preserves the one-to-one spatial relationship between the signals within and observations of the regions is acceptable. To account for such scaling differences, two additional accuracy measures were calculated as in 30 and 31. In 31 κ is a scalar that

(30) $$r^{2}=1-\sum_{\text{elements}}{\left(A^{\text{true}}-\kappa A^{est}\right)}^{2}/{\left(A^{\text{true}}-{\bar{A}}^{\text{true}}\right)}^{2}$$

(31) $$r^{2}=1-\sum_{\text{elements}}{\left(A^{\text{true}}-\text{K}A^{est}\right)}^{2}/{\left(A^{\text{true}}-{\bar{A}}^{\text{true}}\right)}^{2}$$

minimizes the squared difference between the matrices as identified via least-squares. In 3.3, K is a diagonal matrix where each element minimizes the squared difference between corresponding columns of the two matrices.

The simulation matrices were selected to be stable and thus are constrained to have a certain form. Both sDCMf and SLDSf use priors on the A matrices to bias parameter identification toward stable matrices. Thus the “chance” *r*^2^ for the three accuracy measures above given this knowledge is greater than zero. To avoid this bias a final accuracy measure was calculated. Because more than one true transition matrix was created for each simulation (one per condition) the element-wise difference between the true matrices in each simulation was computed (i.e., the change in connectivity from one condition to another). The same difference was computed for the matrices estimated by the connectivity methods. The *r*^2^ between the true difference and the estimated difference was calculated by converting the matrix differences to vectors and correlating them. The expected value of the correlation between the difference matrices is zero whether or not knowledge of the stability of the matrices is used.

In addition, a “chance” accuracy level was determined for the three matrix value accuracy measures. Each simulated transition matrix was used as an “estimate” of all other matrices of the same size with column-wise, global, and no scaling as above. These random estimates were then scaled as appropriate for each of the three measures considered involving the accuracy for a single matrix. This chance measure gives a baseline level of accuracy for a hypothetical method with perfect knowledge of how the system matrices were created. We note that this is more information than any of the methods here had access to and as such may not be a actual baseline but should still be a useful comparison.

## Results

Median *r*^2^ values of the four accuracy measures for each of the three connectivity methods are given in Tables 1**–**4. In general all methods performed above chance levels for all comparisons. All signtests, corrected for multiple comparisons, contrasting the three methods against the chance distributions for all simulation types were significant. Thus, all three methods were able to identify some interregional interactions from the simulated data. All three methods performed best when column-wise scaling was allowed followed by global scaling, then no scaling. All methods were least accurate when examining the differences between system matrices though these accuracies were still superior to chance (zero) levels. Using paired sign tests (Matlab function signtest) to compare median *r*^2^ levels, the sLDSf method achieved superior accuracy to both sDCMf and MAR in all conditions. When column-wise scaling was permitted (Table 1), there were no significant differences in performance between the sDCMf and MAR methods. When only global scaling was permitted (Table 2), sDCMf outperformed MAR for the five-region simulations though there were no significant performance differences between these two methods for the three-region simulations. When no rescaling was permitted (Table 3), sDCMf performance was statistically better than MAR for all simulations except the simplest (two condition, three region) simulations were performance was not reliably different. When considering differences between matrices within simulations, sDCMf performance was statistically better than MAR for all combinations of number of conditions and regions.

**Table 1:** **Median accuracy of estimated transition matrices in *r*^2^**.

**Method**	**Two conditions**	**Three conditions**	**Number of regions**
**COLUMN-WISE SCALING OF ESTIMATED MATRICES**			
sDCMf	0.7631	0.7887	3
	0.6816	0.7059	5
SLDSf	0.8778	0.8879	3
	0.8423	0.8149	5
MAR	0.8158	0.7702	3
	0.6904	0.6888	5

**Table 2:** **Median accuracy of estimated transition matrices in *r*^2^**.

**Method**	**Two conditions**	**Three conditions**	**Number of regions**
**GLOBAL SCALING OF ESTIMATED MATRICES**			
sDCMf	0.6957	0.6817	3
	0.6140	0.6243	5
SLDSf	0.7710	0.7501	3
	0.7316	0.7412	5
MAR	0.7036	0.6404	3
	0.5902	0.6205	5

**Table 3:** **Median accuracy of estimated transition matrices in *r*^2^**.

**Method**	**Two conditions**	**Three conditions**	**Number of regions**
**NO SCALING OF ESTIMATED MATRICES**			
sDCMf	0.5144	0.5179	3
	0.5084	0.5248	5
SLDSf	0.7161	0.7247	3
	0.6852	0.6948	5
MAR	0.5238	0.4523	3
	0.4758	0.4677	5

**Table 4:** **Median accuracy of estimated differences in transition matrices in *r*^2^**.

**Method**	**Two conditions**	**Three conditions**	**Number of regions**
sDCMf	0.3480	0.2436	3
	0.2263	0.1517	5
SLDSf	0.5868	0.4420	3
	0.3971	0.3706	5
MAR	0.2061	0.1052	3
	0.1274	0.1089	5

## Discussion

The current study presents a systematic and valid test between several connectivity methods including the popular sDCMf and MAR. The nature of connectivity estimated by the methods was described at the level of equations between signals of interest. A single equation was found that could accommodate the connectivity type estimated by all three methods. This allowed the three methods to be compared fairly using the same simulated datasets.

While none of the methods performed perfectly, all performed above chance levels. When differential scaling of the signals was accounted for, all methods performed well with most variances-accounted-for exceeding 70%. The more realistic global scaling results were also adequate with all methods performing at approximately *r*^2^ > 0.6. Unfortunately true scaling information would not be available for *in vivo* data. Without scaling the performance of sDCMf and MAR, and to a lesser extent sLDSf, deteriorated. For sDCMf this was most likely due to the use of its own internal scaling which possibly differed from the scaling of the simulation. For MAR this was most likely due to the method's combination of observation noise and signal noise into a single component affecting the signals thus shrinking the connectivity parameters.

As noted however, if prior knowledge of the stability of the transition matrices is used, the expected accuracy of a random matrix is non-zero. The sDCMf and sLDSf methods were able to use this information in the form of priors during estimation. The MAR method applied here used no prior information regarding matrix stability and in addition, required fewer parameters. The reduced performance of MAR relative to sDCMf and sLDSf should be evaluated in light of these facts. Indeed, when column-wise scaling was permitted, performance of MAR was on par with that of sDCMf which used both prior knowledge and multiple additional parameters with a non-linear output function. A Bayesian MAR with appropriate priors would likely improve the performance of the method (Bishop, 2006). However, the QR based regression used here is similar to that used in popular MAR statistical packages (e.g., Schneider and Neumaier, 2001; He et al., 2011; Seth, 2010).

The correlation between the true and estimated task differences, rather than the individual matrices themselves, represents the strongest test of the methods. Prior knowledge of the form of a stable matrix would not improve performance on these differences since deviation from this form (i.e., the condition differences) is random. The sLDSf method performed better than the other methods recovering a median variance accounted for of almost 60% for the simplest condition. However, the remaining accuracies for the other conditions and those for the other methods were fairly poor, dropping to as low as 11% variance-accounted-for by the MAR method. The simulations here used a fairly realistic signal to noise level around 6 db. This corresponds to standard deviations of 0.8 and 0.2 for the signal and noise, respectively, when combined into the observations passed to the connectivity methods. This corresponds to levels seen in somewhat above average quality fMRI data. Higher SNR would likely increase performance of all methods. Given the time commitment required by sDCMf, testing the additional factor of SNR would be difficult. The effect of observation noise magnitude as well as noise type (e.g., white versus colored) on these connectivity methods remains an important area of further study.

The sDCMf and sLDSf methods are quite similar. However, there are three important distinctions between them. First is the inclusion of a probability model for the task in sLDSf that allows for estimation of the task sequence as well as signal states in additional datasets (Smith et al., 2010). Second, sLDSf uses a linear observation equation while sDCMf uses a non-linear system. Third, in estimating parameters, sLDSf uses state estimates conditioned on the full data set; that is, it estimates a probability density for the neural state conditioned on all of the available observed data and uses these estimates to identify parameters. In contrast, sDCMf conditions the probability of the signal state only on the observed data from the beginning of the series up to 25 s after the given time point (Daunizeau et al., 2009). This is because the non-linearity in the output equations makes conditioning on the full series difficult (though see Wan and van der Merwe, 2001). The task probability model contained in sLDSf was not used in the current simulations were it was assumed that the task sequence was known. The superior performance then of sLDSf relative to sDCMf was likely due to the full data conditioning and the relative ease of fitting parameters of linear versus non-linear functions. It is well known that “smoothed” estimates that are conditioned on the full data set are superior to “filtered” estimates that consider only the data up to the current time point. This was shown in an fMRI context using a model similar to sLDSf (Penny et al., 2005). Though the non-linear system output equation used in sDCMf is more flexible and can mimic a greater variety of hIRFs than the linear equation, identifying these parameters requires more complex algorithms. As shown above, the non-linear system model, though based on biophysical realism, is only slightly more accurate than the linear basis method at estimating hIRFs when no non-linearities are present in the signal. For block experimental designs or well spaced event related designs, the cost of this non-linear system model in terms of connectivity parameter accuracy likely exceeds any benefit due to superior estimation of the hIRF. The benefit of the non-linearity would be restricted to situations where stimuli and trials were of variable length and included very short stimuli or where hemodynamic parameters are estimated using data with trials or inputs less than 1–2 s then applied to data with a much longer trials or inputs (Friston et al., 2000). In these cases possible non-linearity in the hIRFs would result in an incorrect response by a linear model. Neither of these situations was applicable in the simulations here thus the non-linearity was not necessary.

**Figure 3:**
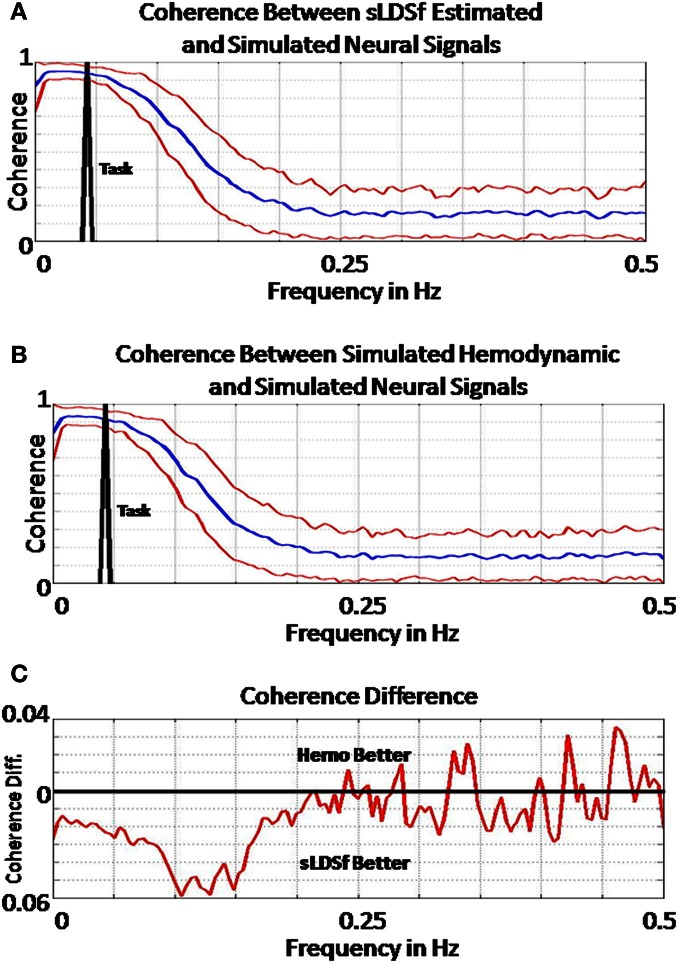
**(A)** Mean (±1.96 SD) coherence estimates between the simulated “neural” signals and those estimated via sLDSf across all two task block simulations. The frequency of task block alternation is shown in black. Coherence between the signals becomes negligible for frequencies above 0.25 Hz. **(B)** A Mean (±1.96 SD) coherence estimates between the simulated hemodynamic and simulated “neural” signals across all two task block simulations. The frequency of task block alternation is again shown in black. As with the estimated data, coherence between the signals becomes negligible for frequencies above 0.25 Hz. **(C)** Mean difference between the coherence estimates for the estimated “neural” signal and the simulated hemodynamic measurements. The zero difference line is shown in black. Values above this line are frequencies where the simulated hemodynamic signal had greater coherence with the simulated “neural” signal than did the estimated “neural” signal. The estimated “neural” signals have marginally better coherence with the true simulated neural signals until approximately 0.2 Hz after which the coherence differences are essentially random.

All of the tested methods were far from perfect at identifying connectivity parameters from the data sets examined here. While higher SNR simulated data would likely increase performance, all of the presented methods suffer from the same flaws that are present in essentially all models of fMRI connectivity. The directed transfer functions of the simulated connectivity matrices considered here contained power at frequencies higher than the Nyquist of typical fMRI sampling rates. Information regarding this portion of the simulated connectivity would be absent in simulated fMRI at realistic sampling rates (cf. Seth et al., 2013). While aliasing of these higher frequency signals is not an issue given that the hIRF passes little below 0.25 Hz (Henson, 2003), the slowness and dispersion of the hemodynamic response creates its own problems. Figure 3A shows the mean (± one standard deviation) coherence between the true signals of interest and those estimated by sLDSf for the two condition, three region simulations. The location of the task transition effect in frequency space is identified in black. Clearly, there is little relation between the true and estimated signals at frequencies higher than 0.2 Hz. Compare Figure 3A with Figure 3B which shows the mean (± one standard deviation) coherence between the true signals of interest and true simulated fMRI data from the same set of data. The difference between the two coherence estimates is shown in Figure 3C where negative values indicate higher coherence for the sLDSf estimate. The coherence between the true signals and the sLDSf estimated signals is slightly better than the simulated fMRI for lower frequency portions of the data. However, due to the hemodynamic filtering, neither the simulated fMRI data nor the SLDSf estimate of the signal accurately approximates the high frequency portions of the true signal of interest.

Unfortunately, the higher frequency components of the true signal are simply not contained in the measured fMRI data when examined as a time series. Though a proper experimental design can be used to identify sub-second differences in BOLD signal onsets, data sampled at 1 Hz. cannot contain information regarding signals with frequencies higher than 0.5 Hz. None of the methods examined here can be expected to recover information about a signal that is not there to recover. None of the methods examined here will truly identify interactions occurring between regions a few hundreds of milliseconds apart unless the interaction produces longer lasting consequences. Temporal directionality of individual neural “spikes” 100 ms. Apart in time cannot be seen using fMRI data at conventional sampling rates. However, lower frequency fluctuations in the power of high frequency signals may be observed and are likely more relevant to connectivity analysis in fMRI (cf., Martínez-Monts et al., 2004). MAR models and linear dynamic systems can be indentified within limited frequency bands (Wills et al., 2009). Such frequency restrictions could force the methods to ignore higher frequency components of the signal that could not possibly be passed through the hemodynamic filter. This would have the added benefit of encouraging researchers who use connectivity methods to consider the meaning of the identified connectivity parameters from a signal processing stand-point rather than simply considering the numeric value of the parameters in isolation (Smith et al., 2011b).

Based on the overview presented here, we make the following recommendations for fMRI connectivity simulation studies. While simulation based studies of connectivity methods are necessary, they are not trivial to perform. Some parts of some models are directly comparable after appropriate conversion. However, some models will always remain distinct. For example, deterministic DCM cannot be used to simulate data for SEM; one considers the task effect on internal dynamics (changes in A) in the absence of state noise while the other considers the task effect on state noise patterns (changes in G) in the absence of internal dynamics. Comparisons of methods that ignore this issue as well as issues concerning the distinction between continuous and discrete time can lead to false conclusions (cf. Smith et al., 2011a). In addition, simulation studies that fail to consider signal processing realities relevant to fMRI (for example by simulating then attempting to recover directionality of individual neural spikes separated by a few tens of milliseconds) can also lead to incorrect conclusions. For all simulations then, care must be taken to identify the nature of the connectivity simulated by a model as well as the extent to which that type of connectivity is present in the actual data simulated.

Based on the results presented here, we make the following recommendations for fMRI connectivity analyses. For exploratory analyses, unless non-linearities in the hIRFs are expected to be relevant, sDCMf is over-parameterized, slow, and less accurate and is thus not recommended. SLDSf outperformed MAR models but at a cost of additional parameters. For small models (i.e., less than 10 regions) the additional parameters of SLDSf relative to MAR may not be of concern and SLDSf is recommended. However, for larger models where the number of parameters would approach the degrees of freedom of the data, MAR with appropriate priors may be preferred. Because of the speed of estimating parameters in MAR models, this preference extends to full brain, seed region analyses where hundreds of thousands of models are identified such as Granger Causal Mapping (Roebroeck et al., 2005). For confirmatory models where certain connections are assumed to take known values (e.g., 0), estimating a model in continuous time may be preferable. The conversion from continuous to discrete time using Equation 17 for models with extensive feedback can cause connections “known” to be zero in continuous time to be non-zero when considered in discrete time. In such cases, there will be multiple continuous time models that are all equally compatible with the discrete time data and a priori knowledge must be used to select one over the others. To account for known parameter values defined in continuous time, the models can be identified in continuous time (as in sDCMf) or identified in discrete time and a second optimization applied to convert this model to continuous time appropriate known zeros (Ljung and Wills, 2010). It remains to be seen if there is a preference for either of these methods in fMRI.

## Conclusion

Connectivity, though widely used, has not always been well-defined. Comparing methods that use divergent underlying connectivity equations using a single simulated data set can be problematic. Here we compared the performance of three methods with a single common connectivity equation. Performance of all methods was better than chance though no method could capture high frequency components of the data. The results here, as well as basic signal processing theory, suggest that fMRI researchers should avoid searching for interregional interactions at frequencies higher than those passed by the hemodynamic filter. Researchers should also avoid interpreting fMRI connectivity results in terms of single event communications between regions on timescales of tens or even hundreds of milliseconds.

### Conflict of interest statement

The authors declare that the research was conducted in the absence of any commercial or financial relationships that could be construed as a potential conflict of interest.

## Footnotes

^1^We use the tilde ~ to indicate “distributed as” and *N*(*a*, *b*) to indicate a normal distribution with mean a and covariance (inverse precision) *b*.

^2^The limit being taken as σ and thus *R* go to zero rather than simply setting *R* to zero is only necessary to preserve a proper probability model for PCA (Tipping and Bishop, 1999).

^3^This is, however, a considerable abuse of the terminology of ICA. Typically ICA would refer to ε as the underlying signals or sources and, since *R* is 0, would not distinguish between observations Y and X. Despite the odd terminology used the equations are correct.

^4^These assumptions lead to recursive SEMs (i.e., a SEM that is described by a directed acyclic graph) that are uniquely identifiable via ordinary least squares. Some cyclic (i.e., non-recursive) SEMs can be identified using instrumental variables or other approaches, but identification can be considered only on a case by case basis. Without instrumental variables, often only a set of multiple equivalent non-recursive (cyclic) models can be identified without resorting to stochastic sampling methods (Richardson, 1996; Storkey et al., 2007; Lacerda et al., 2008).

^5^Also referred to as vector autoregressive (VAR) and, if instantaneous relationships between the signals are considered, structural vector autoregressive (SVAR) models.

^6^The R matrix could also be set to zero to ensure a pure MAR model; otherwise 1.2 and 1.10 describe a similar but more general state space model (Lütkepohl, 2007). Any MAR model can be described in state space form, but not all state space models are MAR models.

^7^Strictly speaking a MAR analysis is not identical to a Granger Causality analysis though the terms are often used interchangeably. See REFS for more details.

^8^Most descriptions of bilinear models for connectivity use B to indicate the bilinear term that is labeled H here.

^9^If there are known zeros in *G*, it can be solved via maximum likelihood methods rather than Cholesky factors.

^10^While the discrete time error covariance matrix Q is unique, the representation of the same system using the *G* matrix may not be. There may be additional *G* matrices for which GIG^*T*^ = Q. This is not a concern if a method solves for *Q* rather than *G*.

^11^The MAR regressions were also attempted using the inputs at the true time convolved with a canonical hIRF. This resulted in poorer performance as did deconvolution of the observations using a canonical hIRF (division in frequency space followed by a low-pass filter). Results of these inferior methods are not reported here.

## Appendix

The discrete time dynamic system can be described using the temporal shift-operator, *q* as in A1:

(A1) $$qx_{t}=Ax_{t}+Bu_{t}+\varepsilon_{t}$$

where,

(A2) $$qx_{t}\equiv x_{t\;+1}$$

The delta-operator, δ can be defined in terms of the shift-operator:

(A3) $$\begin{array}{l} \;\delta ≜\frac{q-1}{\Delta }\\ \delta x_{t}=\frac{(q-1)x_{t}}{\Delta }\\ \delta x_{t}=\frac{x_{t\;+1}-x_{t}}{\Delta } \end{array}$$

where Δ is the step size. The same discrete time dynamic system can also be described using the delta operator:

(A4) $$\delta x_{t}=Ex_{t}+Fu_{t}+\xi$$

Collecting terms of *x_*t*_* yields:

(A5) $$\delta x_{t}-Ex_{t}=Fu_{t}+\xi$$

Using the definition of the delta-operator and the shift-operator yields,

(A6) $$\begin{array}{l} \;\frac{(q-1)x_{t}}{\Delta }-Ex_{t}=Fu_{t}+\xi \\ \;\frac{qx_{t}-x_{t}}{\Delta }-Ex_{t}=Fu_{t}+\xi \\ \Delta^{-1}x_{t\;+1}-\Delta^{-1}x_{t}-Ex_{t}=Fu_{t}+\xi \\ \;x_{t+1}-(I+E\Delta )x_{t}=\Delta (Fu_{t}+\xi )\\ \;x_{t+1}=(I+E\Delta )x_{t}+\Delta Fu_{t}+\Delta \xi \end{array}$$

Thus,

(A7) $$\begin{array}{l} A=(I+E\Delta )\\ B=F\Delta \end{array}$$

Equation A6 shows the utility of the delta-operator form. If the sampling rate is very fast relative to the dynamics of the system, EΔ approaches zero and A approaches I.
